# Optical pH Sensor Based on Immobilization Anthocyanin from *Dioscorea alata* L. onto Polyelectrolyte Complex Pectin–Chitosan Membrane for a Determination Method of Salivary pH

**DOI:** 10.3390/polym13081276

**Published:** 2021-04-14

**Authors:** Eka Safitri, Hani Humaira, Murniana Murniana, Nazaruddin Nazaruddin, Muhammad Iqhrammullah, Nor Diyana Md Sani, Chakavak Esmaeili, Susilawati Susilawati, Muhammad Mahathir, Salsabilla Latansa Nazaruddin

**Affiliations:** 1 Department of Chemistry, Faculty of Mathematics and Natural Sciences, Universitas Syiah Kuala, Banda Aceh 23111, Indonesia; hanihum18@gmail.com (H.H.); murniana@unsyiah.ac.id (M.M.); nazaruddin.ma@unsyiah.ac.id (N.N.); susilawatisulaiman64@yahoo.com (S.S.); 2 Graduate School of Mathematics and Applied Sciences, Universitas Syiah Kuala, Banda Aceh 23111, Indonesia; m.iqhram@oia.unsyiah.ac.id; 3 Sanichem Resources Sdn. Bhd., No 7/7A, Jalan Timur 6/1A, Mercato Enstek, Bandar Estek 71060, Negeri Sembilan, Malaysia; diyanasani@yahoo.com; 4 Center of Excellence in Electrochemistry, Faculty of Chemistry, University of Tehran, Tehran 4176-14411, Iran; chakavak486@yahoo.com; 5 Faculty of Dentistry, Universitas Syiah Kuala, Banda Aceh 23111, Indonesia; cakothir@gmail.com (M.M.); salsanazaruddin@gmail.com (S.L.N.)

**Keywords:** optical pH sensor, anthocyanin, *Dioscorea alata* L., polyelectrolyte pectin–chitosan, saliva

## Abstract

A simple optical pH sensor based on immobilization, *Dioscorea alata* L. anthocyanin methanol extract, onto a pectin–chitosan polyelectrolyte complex (pectin–chitosan PEC), has been successfully fabricated. The optical pH sensor was manufactured as a membrane made of pectin–chitosan PEC and the extracted anthocyanin. This sensor has the highest sensitivity of anthocyanin content at 0.025 mg/L in phosphate buffer and 0.0375 mg/L in citrate buffer. It also has good reproducibility with a relative standard deviation (%RSD) of 7.7%, and gives a stable response at time values greater than 5 min from exposure in a buffer solution, and the sensor can be utilized within five days from its synthesis. This optical pH sensor has been employed to determine saliva pH of people of different ages and showed no significant difference when compared to a potentiometric method.

## 1. Introduction

Pectin and chitosan are natural polymers that have been widely used in the food industry, medicine, and the environment [1,2,3,4,5,6]. Membranes made of the two polymers have also been applied as a matrix in the development of sensors and biosensors. In regards to sensor and biosensor development, pectin and chitosan have been used as matrices for the binding of active substances, which will interact with analytes or other ingredients added, to improve the performance of sensors or biosensors. In this case, compatibility between the matrix and the active substance plays an important role in terms of sensitivity and excellent response time [7]. In many cases, synthetic polymer matrices possess several advantages, such as excellent mechanical strength, temperature resistance, and plasticity [8]. Frequently, synthetic polymer matrices are hydrophobic and are very suitable for sensors or biosensors that require immersion in aqueous media, such as voltammetry and potentiometry methods. Accordingly, such hydrophobic polymer membranes will not easily leach out from the matrices into the sample or solution, hence minimizing lost in sensor sensitivity. However, the hydrophobic membrane causes a slow diffusion process between active material and the analyte [9], resulting in a slow response.

Pectin and chitosan, taken individually, have many weaknesses [10,11]. Pectin is soluble in water, while chitosan is very soluble in an aqueous environment at low pH [8,12]. Some sensors have been fabricated based on pectin and chitosan membranes. The use of pectin in the development of optical pH sensors with less volume of the sample has been previous reported [13,14]. Pectin membrane possesses hydrogel properties, and it is not appropriate to be used for the immersion of such sensors in samples during analysis. At the same time, a number of studies on chitosan-based sensors have also been reported [15,16,17]. Therefore, efforts to combine both polymers have already started with the formation of a polyelectrolyte complex (PEC) membrane. In this particular case, two polyelectrolytes (polymers carrying ionizable functional groups as side-substituents anchored along their backbones) of opposite charges interact favorably to give an interpolymeric complex, PEC [18,19,20,21,22]. The modification of pectin and chitosan into a pectin–chitosan PEC membrane has good mechanical strength. PEC membranes are formed through electrostatic interactions between the carboxylate groups (-COO^−^) in the pectin and the primary ammonium groups (-NH_3_^+^) in chitosan. Chitosan acts as a polycation in acidic solutions, and pectin acts as a polyanion after it is dissolved in water. The formation of a polyelectrolyte complex pectin–chitosan may be induced most likely by attractive electrostatic interactions between the two macromolecular [15].

In this study, a chitosan-pectin PEC membrane has been obtained and used as a matrix to develop an optical pH sensor by immobilizing the anthocyanin-a pH sensitive compound extracted from the *Dioscorea alata* L. Anthocyanin is capable of displaying different colors over a wide pH range and, therefore, is suitable to be employed as an active pH substance for optical measurement [16,17]. Moreover, all of the materials used in the construction of the optical pH sensor are non-toxic, hence it is safe to use for in situ analysis. Indeed, a similar PEC system with immobilized anthocyanin has been employed to construct a pH indicator [18]. However, in the aforementioned study, the performance was analyzed based on the change of the color of anthocyanin at different pH values using a colorimeter, hence the analysis was only qualitative. In contrast, our work performed a qualitative approach using UV–Vis spectrophotometer to investigate and optimize the sensitivity, response time, linear range, reproducibility, and response stability of the fabricated sensor. Additionally, during the construction of our optical pH sensor, the selection of the composition ratio was based on the one that could yield a transparent membrane as opposed to the previous study [18] that was based on the stoichiometric calculation. This sensor was then utilized to determine the acidity of human saliva and is able to work for a small amount of sample. Generally, an analytical method, such as determining the pH of saliva using a pH ion selective electrode (ISE), requires a larger sample volume. Large volumes of saliva can be difficult to obtain, so analytical techniques that require large numbers of samples are not suitable for this kind of analysis. The usefulness of the sensor described herein relies on the fact that the examination of saliva is a routine analysis especially for dental and oral health assessment [19,20,21].

## 2. Chemicals and Apparatus

Chemicals used in this research, such as monopotassium phosphate (KH_2_PO_4_) and dipotassium phosphate (K_2_HPO_4_) were acquired from Fluka (Steinhem, Germany); and pectin and methanol from Sigma (St Louis, MO, USA).

Chitosan was acquired from Tokyo Chemical Industry Co., Ltd., Japan, obtained from shrimp shell, with a deacetylation degree of 75–85%. *Dioscorea alata* L. was purchased from a local market; HCl, NaOH, citrate acid, sodium citrate, pectin, acetic acid from Merck (Darmstadt, Germany); saliva from volunteers.

Absorbance measurements associated to the response of the biosensor were performed by spectrophotometer UV–Vis Shimadzu 1800 (Kyoto, Japan); buffer pH was monitored with a pH-meter Thermo Scientific Orion Star A211 (Waltham, MA, USA). Pectin membrane surface morphology was analyzed employing a Zeiss Merlin/Merlin Compact/Supra 55VP Field Emission Scanning Electron Microscope (FESEM) (Carl Zeiss, Jena, Germany) at an acceleration voltage of 10kV and a magnification of 4 kx. Investigation on structural and thermal properties of the obtained systems was performed by IR spectroscopy (FTIR) Cary 630 Anti Agilent instrument (Santa Clara, CA, USA). Shimadzu DTG-60 thermal gravimetric analyzer (DTG) and Shimadzu DSC-60 differential scanning calorimetry (DSC) (Shimadzu, Kyoto, Japan), respectively.

## 3. Experimental

### 3.1. Preparation of Anthocyanin/Pectin–Chitosan

**Figure 1:**
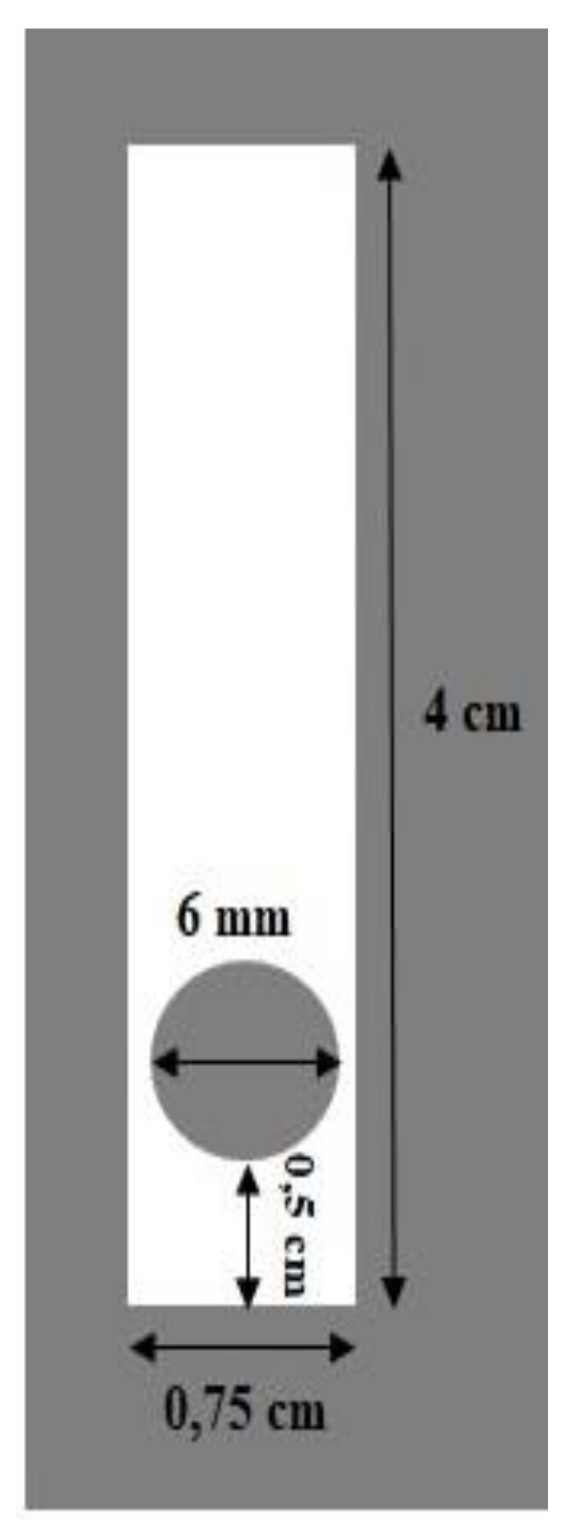
Optical pH sensor design.

Anthocyanin was extracted from *Dioscorea alata* L. using methanol solvent following a previously reported procedure [22]. Pectin–Chitosan PEC membrane was prepared by mixing each pectin solution and chitosan 1% (*w/v*) in a weight ratio of 3:7. Pectin and chitosan were dissolved using distilled water and 4 M CH_3_COOH to the final volume of 100 mL The mixture was stirred until homogeneous. The pectin–chitosan PEC solution was mixed with anthocyanin from its stock extract of 0.150 mg/L to obtain PEC solutions containing anthocyanin at concentrations of 0.0250, 0.0375, and 0.050 mg/L. A sample of 30 µL PEC-anthocyanin mixture was dripped onto a circle with a diameter of 6 mm onto a 4 × 0.75 cm mica plastic as illustrated in Figure 1 and dried in a refrigerator at 4 °C for 24 h.

### 3.2. Membrane Characterization

Pectin, chitosan, and PEC membrane functional groups were analyzed using a Fourier-transform-infrared spectroscopy (FTIR). IR spectra were acquired in the range of 4000–650 cm^−1^.

As for the membrane’s surface morphology, it was investigated by employing a scanning electron microscope with the following operating parameters: Accelerating voltage 10 kV and magnification of 4 kx with the following parameters: Voltage = 10 kV, distance = 25 mm, current intensity = 200 pA, and vacuum pressure = 10^−5^ Torr (1.3 × 10^−3^ Pa).

A sample, as much as 1 mg, was placed on an aluminum crucible and heated from 50 °C to 600 °C at 40 °C/min under dynamic nitrogen atmosphere (flow rate: 20 mL/min) in a Shimadzu DTG-60 Thermal Gravimetric Analyzer. DSC thermograms were acquired on a DSC instrument under nitrogen atmosphere (flow rate—20 mL/min) and temperature range of 40 °C to 600 °C (40 °C/min).

### 3.3. Sensitivity Optical pH Sensor Determination in Citrate and Phosphate Buffer Solutions

The sensitivity of optical pH sensor was determined based on measuring absorbance in citrate (pH 4–8), and phosphate (pH 4–9) buffer solutions at each wavelength of maximum absorbance. Thus, volumes of 30 µL, taken individually, of 0.1 M buffer solution (citrate and phosphate) with a certain pH, were dropped onto the pectin/chitosan/anthocyanin membrane (optical pH sensor). Afterwards, the measurements were performed, taking into account the maximum absorbance values exhibited by anthocyanin. The measuring of absorbance was carried out 10 min after dropping 30 µL of buffer solution at various pHs. Dependences between the absorbance and pH values on different pH ranges were plotted to obtain the corresponding dynamic ranges. The calculated slopes of these linearly fitted dependencies are considered the sensor sensitivity values.

### 3.4. Determination of Sensitivity of Optical pH Sensors at Various Phosphate Buffer Concentrations

The buffer solution, which showed the highest sensitivity value, was changed in its concentration (0.01, 0.03, 0.05, and 0.1 M) to get the maximum sensitivity within a wide pH range.

### 3.5. Reproducibility, Response Time, and Lifetime Studies on the Optical pH Sensor

The response time was determined by measuring the response of sensors at 5, 10, 15, 20, 25, and 30 min after dripping 30 µL samples of various pHs of the buffer solutions (with optimum concentration) onto the sensor surface. The reproducibility was assessed based on absorbance measurements of 10 sensors by adding 30 µL of 0.1 M of PBS at pH 7. The lifetime was studied by measuring the absorbance of sensor at different days (1, 2, 3, 4, 5, 10, 15, and 20).

### 3.6. Real Sample Measurement

A real sample study was carried out on the saliva pH testing. About 30 µL of saliva from each volunteer was dropped on the surface of the membrane sensor, and absorbance was measured after 5 min. The absorbance value obtained was then converted to a pH value using a linear equation obtained from a corresponding calibration curve.

## 4. Results and Discussion

### 4.1. Synthesis and FTIR Characterization of the Investigated Systems

As mentioned above, pectin–chitosan PEC membrane is mainly a result of attractive electrostatic interactions between the two weak polyelectrolytes, one of them being polyanion (pectin) and the other one (chitosan)-polycation.

During PEC formation, the flexibility of each type of macromolecular chain is progressively reduced and, concomitantly, all the hydrophilic ionizable groups are pointed towards each other in a pairwise manner (carboxylate groups to primary ammonium ones), separated from the aqueous environment. As a result of these salt-like bridges established between the two kinds of oppositely charged groups, pectin–chitosan PEC membrane become practically insoluble in aqueous medium. At the same time, the interaction between the two polymers pectin and chitosan will be able to improve the mechanical characteristic of the pectin–chitosan PEC membrane produced [15].

**Figure 2:**
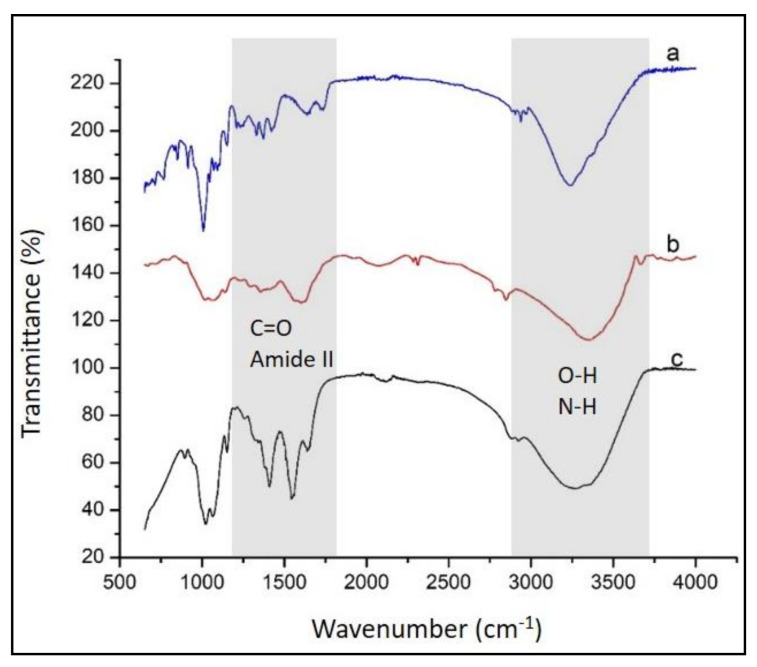
FTIR spectra acquired for membranes of (**a**) pectin, (**b**) chitosan, and (**c**) pectin–chitosan PEC (3:7).

In this study, membranes with a composition expressed as weight ratio between pectin and chitosan of 3:7 were manufactured. These PEC membranes exhibited a homogeneous surface and a good transparency. The results of the study show that the composition with chitosan exceeding this optimum ratio led to a transparent membrane, but rigid and brittle. Furthermore, pectin–chitosan PEC membranes containing pectin levels are higher than the specified level yielded pectin–chitosan PEC membranes that are less homogenous and with a great availability to gel forming in water. That is why the specified composition of the pectin–chitosan PEC membranes was selected for manufacturing the pH sensor matrix. Structural characterization of pectin, chitosan, and PEC (3:7) membranes was carried out by FTIR spectroscopy and the results were graphically collected in Figure 2.

Based on the FTIR spectroscopy data, the presence of the functional moieties belonging to pectin (Figure 2a) were revealed by the IR vibrations at 1597, 1716, and 3232 cm^-1^ assigned to carboxylate (—COO—), carbonyl (C=O) from ester and hydroxyl (intra- and intermolecular hydrogen bonding) vibrations, respectively [1,23]. FTIR spectrum of chitosan (Figure 2b) shows the presence of C=O stretching at 1642 cm^−1^ associated to the remaining acetyl moieties (amide I), a vibration band located at 1564 cm^−1^ assigned to N—H bending of amine groups, a vibration band at 1320 cm^−1^ (amide III), skeletal vibrations involving C—O stretching around 1000 cm^−1^, and a broad band at 3450 cm^−1^ ascribed to O—H stretching mixed with N—H stretching vibrations of amide groups [24,25]. The analysis of IR spectrum of pectin–chitosan PEC membrane moieties show a sharp peak at 1566 cm^-1^ assigned to an amide II band. On the other hand, in the PEC membrane, it is difficult to individually identify the presence of carboxylate groups of pectin (involved in the attractive interactions between the two oppositely charged polyelectrolytes) because their IR vibrations overlap the vibrations of C=O stretching of the remaining acetyl groups of chitosan. Thus, the band located near 1640 cm^−1^ in the PEC membrane can be ascribed to the common contribution of the two types of functionalities just mentioned.

**Figure 3:**
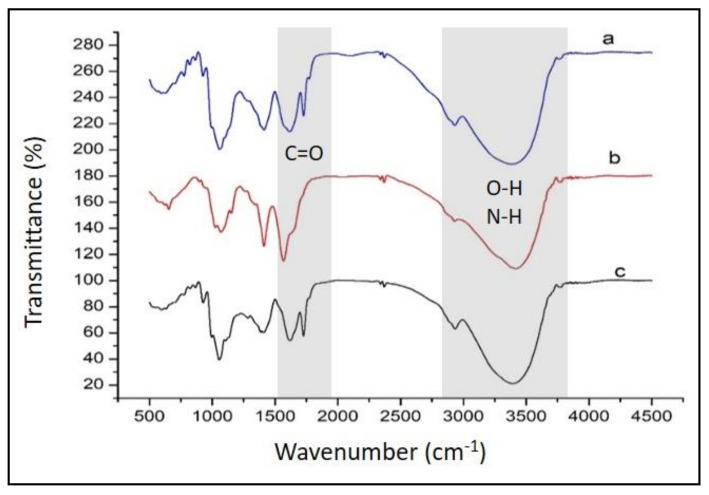
FTIR spectra of (**a**) anthocyanin, (**b**) pectin–chitosan, and (**c**) anthocyanin/pectin–chitosan/ membranes.

FTIR spectra of anthocyanin extracted from *Dioscorea alata,* by comparison with those of the membranes made of pectin–chitosan and anthocyanin pectin–chitosan, are shown in Figure 3. The main IR spectral features characteristic to anthocyanin dye consist of a broad band at around 3500 cm^−1^ attributed to OH groups responsible for hydrogen bonding, a band at ca. 1750 cm^−1^ representing C=O stretching vibrations, a region between 1500 and 1700 cm^−1^ assigned to C=C stretches of aromatic moieties, and a band located at around 1000 cm^−1^ associated with C—O—C (ether) stretching vibrations [26]. By comparing IR profiles in Figure 3, it is obvious the presence of anthocyanin within the pectin–chitosan PEC membrane (IR spectrum c).

### 4.2. Morphological Properties

**Figure 4:**
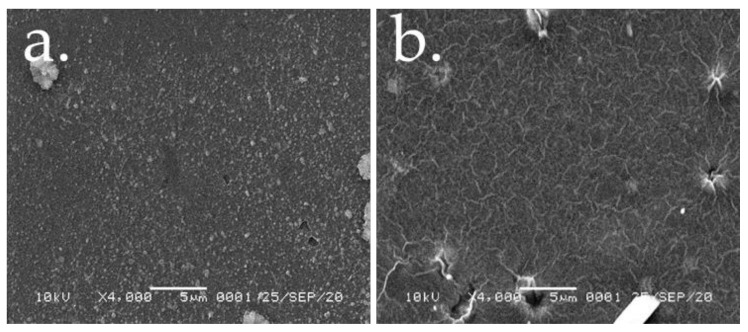
Morphologies of (**a**) pectin–chitosan, and (**b**) anthocyanin/pectin–chitosan membranes revealed by SEM.

The morphological properties of the PEC membrane were revealed by SEM. To a high electric conductivity in order to avoid any damages under the action of the electron beam, all the samples were firstly coated by a thin Pt layer. Thus, the obtained morphologies for PEC (3:7) membranes (with and without anthocyanin) are presented in Figure 4. The SEM images show relatively smooth, homogeneous membrane surface, even though the superficial texture appears to be slightly changed for the membrane with anthocyanin.

### 4.3. Thermal Characteristics

**Figure 5:**
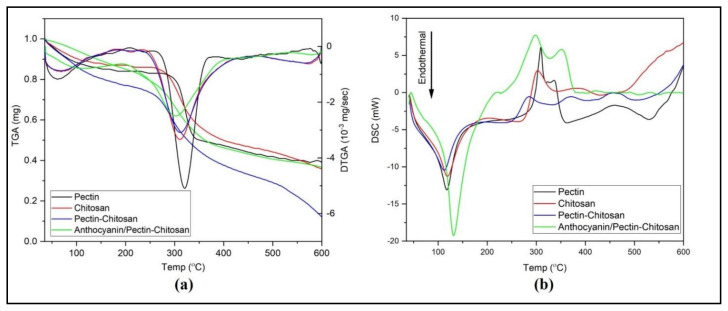
Thermal characteristics of pectin, chitosan, and pectin–chitosan as shown by (**a**) TGA/DTGA and (**b**) differential scanning calorimetry (DSC) thermograms.

Thermal behavior of the manufactured PEC membranes was investigated by thermogravimetric analysis (TGA) performed on a temperature range of 35–600 °C (Figure 5a). Initial broad minimum located on derrivative TGA (DTGA) curves at T_peak_ of 58 °C is attributed to water loss moisture retained due to the membranes’ hydrophilicity [27]. More significant weight loss occurs within the temperature ranges of about 235–435 °C for chitosan and pectin–chitosan and around 260–373 °C for pectin, suggesting a process of polymer degradation/decomposition. TGA/DTG data are roughly confirmed by those of differential scanning calorimetry (DSC) obtained on the same samples (Figure 5b).

In DSC experiments, the loss of water can be observed by a slight endothermic shoulder before the temperature reaches 120 °C was observed in all samples similar with the TGA profile. However, a sharper endothermic peak observed at higher temperature (ca. 120 °C peak temperature) in anthocyanin/pectin–chitosan indicates a moisture content strongly retained/bound to the polymer matrix of the studied systems. Similar to other DSC data reported elsewhere [28,29] a number of exothermic processes located at peak temperatures of 281 °C, 298 °C, 309 °C, and 303 °C were assigned to the polymer chain decomposition in pectin–chitosan, anthocyanin/pectin–chitosan, chitosan and pectin membranes, respectively.

Due to the weight composition of PEC systems richer in chitosan, both DTGA and DSC traces of the investigated PEC membranes roughly exhibit a resemblance closer to the chitosan membrane than that of pectin, as expected, and as can be seen in Figure 5.

### 4.4. Optical pH Sensor Optimization

#### Effect of the Anthocyanin Concentration on Sensor Sensitivity

The effect of anthocyanin content as an active and pH-sensitive compound on sensor sensitivity was studied. The corresponding absorbance values of the sensor membranes prepared with the three different concentrations of anthocyanin prior to membrane formation (0.0250, 0.0375, and 0.0500 mg/mL) were measured after the addition of 0.1M buffer solution (both citrate and phosphate buffer of various pHs) and the results of sensitivity determination are collected in Table 1. The best sensitivity for citrate buffer was at concentration of 0.0375 mg/L within pH range 4.0–5.5 and sensitivity value of 0.0795 (R^2^ = 0.9985). The highest sensitivity in phosphate buffer was found at a concentration of 0.025 mg/L with a pH range of 4.8–9.0 and a sensitivity value of 0.0785 (R^2^ = 0.983). The optimum anthocyanin concentration obtained was used for further characterization.

Table 1 shows that the optical pH sensor has a narrower dynamic range in citrate buffer solution compared to that in phosphate buffer solution. This is because the pKa values of the conjugate acid-base pairs associated to phosphoric acid (involved in phosphate buffer) cover a broader buffer range than in the case of citric acid (involved in citrate buffer). A higher acidity of the environment (like in citrate buffer) causes the color of anthocyanin to fade, leading to low absorbance and, as a consequence, low sensitivity of the sensor. Even though citrate buffer yielded slightly higher sensitivity at a pH of 4.0–5.5 and anthocyanin concentration of 0.0375 mg/L, the applicability might be limited by its narrow pH range. Therefore, PEC sensor in phosphate buffer with anthocyanin concentration of 0.025 mg/L was selected for further studies.

Table 2 shows the effect of phosphate buffer concentration on the sensitivity of the pH optical sensor. The results indicate that 0.1M phosphate buffer solution led to the best results with a sensitivity value of 0.0786 and a coefficient of determination (R^2^) of 0.9838.

### 4.5. Characterization of Optical pH Sensor

#### 4.5.1. Response Time

**Figure 6:**
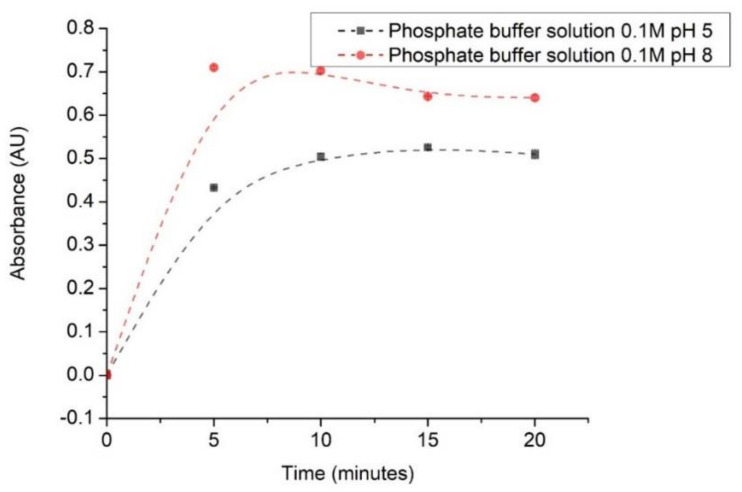
Response time profile of the selected (most sensitive) optical pH sensor.

Response time was determined at 0, 5, 10, 15 20, 25, and 30 min in 0.1M phosphate buffer solution of pH 5 and pH 8, and the data are plotted in Figure 6.

The increase in absorbance during the first 5 min of measurement is caused by the reaction time required by anthocyanin and the buffer solution. Then, absorbance values tend to make a plateau due to the stability of the anthocyanin color after the reaction time [23]. It is worth mentioning that the reaction may take less than 5 min. However, the stable response was only obtained afterward. Indeed, paper-based universal pH indicator may yield faster response due to its hydrophilic properties. However, such pH indicator has been reported to produce poor quantitative data [30].

#### 4.5.2. Reproducibility of Optical pH Sensors

The reproducibility of the sensor was studied to evaluate the performance of the sensor by measuring the absorbance of ten different sensors of the same type under the same experimental conditions. The results obtained are shown in Table 3.

Table 3 shows that the absorbance value for the 10 optical pH sensors averaged at 0.458 with a standard deviation of 0.035 and a percentage relative standard deviation (% RSD) of around 7.69%. The results indicate that this sensor meets the criteria for reproducibility in terms of good standard deviation and percentage RSD.

#### 4.5.3. Lifetime Profile of Optical Sensor pH

**Figure 7:**
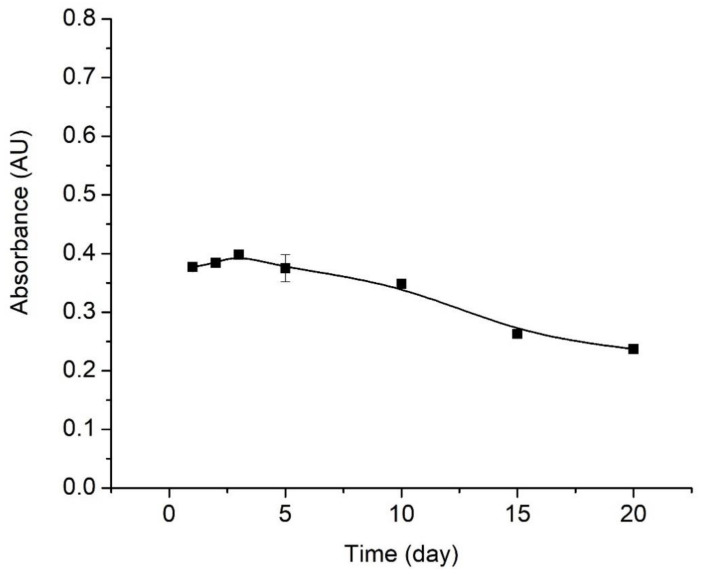
Lifetime profile of optical pH sensor.

The sensitivity of the sensor can decrease after prolonged storage and use. Determination of the lifetime of the pH optical sensor was evaluated by measuring the absorbance of the sensor on different days and carried out for 20 days. Results are shown in Figure 7.

The lifetime study was carried out over a time period of 20 days, with absorbance measurements performed after 1, 2, 3, 5, 10, 15, and 20 days of sensor storage. According to the test results, the pH optical sensor gives a good performance from day 1 to day 3 based on the increase in absorbance. However, the measurements conducted on the 5th to 20th days showed reduced sensor responses as observed by decreasing absorbance values. Essentially, by comparison to the signal recorded on the first day, the percentage decrease in sensor signal is of 2.9% on the 10th day, 11.4% on the 15th day, and 14% on the 20th day.

## 5. Salivary pH Determination

The optimized sensor was then tested on human saliva samples. The saliva samples were categorized according to age and the before and after meals in the span of 1 h. The pH values of saliva were determined based on the absorbance measurements, which were then converted to pHs according to a calibration curve. The results of testing saliva samples are shown in Table 4.

Determination of saliva pH was also carried out on pregnant women before and after meals within 1 h. Saliva samples were taken every 15 min after each meal. The results of before and after meal saliva tests are collected in Table 5.

The data from Table 4 and Table 5 show that salivary pH measured by optical sensors is not significantly different from pH measured by ISE H^+^.

Based on t-test calculations, the obtained t-values were lower than that of t-table. It means the two methods for measuring salivary pH were not significantly different. In addition, normal pH in the mouth is 7. Salivary acidity (pH) is one of the important factors that favor dental caries, periodontal abnormalities, and other diseases of the oral cavity. At the same time, bacterial growth on teeth is optimum at salivary pH from 6.5–7.5. On the other hand, low salivary pH (4.5–5.5) will facilitate the growth of acidogenic germs such as *Streptococcus mutants* and *Lactobacillus* [20,31,32]. All of these aspects, abovementioned, show the importance of easy detection of intraoral pH by using very small saliva samples and employing an appropriate sensor.

## 6. Conclusions

Anthocyanin from the tuber of *Dioscorea alata* L. has been used as a pH-sensitive active component, and it has been successfully immobilized onto a pectin–chitosan PEC membrane for designing an optical pH sensor. The formation of PEC from polyanionic pectin and polycationic chitosan was successfully carried out and confirmed by FTIR spectroscopy. Thermal of the anthocyanin/pectin–chitosan reveals a good stability of the optical pH sensor in over a large range of temperature. Analytical performance and its capacity to give a reliable response on small sample volumes within a wide range of pHs make the optical pH sensor very useful in measuring human saliva pH.

## Figures and Tables

**Table 1:** Sensitivity of the PEC sensor against various anthocyanin concentrations in 0.1M citrate and phosphate buffers.

Anthocyanin Concentration (mg/L)	Citrate Buffer			Phosphate Buffer		
	pH Range	Sensitivity (AU/pH Unit)	R^2^	pH Range	Sensitivity (AU/pH Unit)	R^2^
0.025	4.0–6.0	0.0423 ± 0.003	0.933	4.8–9.0	0.0785 ± 0.001	0.9830
0.0375	4.0–5.5	0.0795 ± 0.004	0.999	4.8–9.0	0.0744 ± 0.001	0.9679
0.05	5.0–7.5	0.0128 ± 0.001	0.969	4.8–7.5	0.0730 ± 0.003	0.9711

**Table 2:** Sensitivity of optical pH sensor in phosphate buffer solution of different concentrations.

Phosphate Buffer			
Concentration (M)	pH Range	Sensitivity (AU/pH Unit)	R^2^
0.01	7.0–9.5	0.0588 ± 0.0145	0.9760
0.03	7.0–9.5	0.0623 ± 0.0070	0.9616
0.05	6.5–9.5	0.0682 ± 0.009	0.9614
0.075	4.8–9.5	0.056 ± 0.02	0.9746
0.1	4.8–9.5	0.0786 ± 0.001	0.9838

**Table 3:** Reproducibility test for the optical pH sensor.

No.	Optical pH Sensor	Absorbance (AU)
1	Sensor A	0.461
2	Sensor B	0.450
3	Sensor C	0.401
4	Sensor D	0.413
5	Sensor E	0.460
6	Sensor F	0.463
7	Sensor G	0.513
8	Sensor H	0.471
9	Sensor I	0.436
10	Sensor J	0.516
Average		0.458
SD		0.0352
RSD (%)		7.687

SD = standard deviation; RSD = relative standard deviation.

**Table 4:** Salivary pH determination from three peoples.

No.	Age of People from Whom Saliva Samples Were Tested before Meals (Years)	pH Determined by Optical pH Sensor	pH Determined by Ion Selective Electrode (ISE H^+^)	T_value_	T_table_
1.	6	7.23	7.3	0.835	2.92
2.	22	6.83	7.15	2.29	
3.	56	6.50	6.8	1.42	

**Table 5:** pH determination on saliva samples from pregnant women before and after meal.

No.	Time after Meal (Min)	Optical pH Sensor	ISE H^+^	T_value_	T_table_
1.	Before meal	6.83	7.15	2.29	2.92
2.	15	6.76	6.73	1.48	
3.	30	6.62	6.63	0.539	
4.	45	6.85	6.81	1.09	
5.	60	6.96	6.91	1.80	

## Data Availability

No new data were created or analyzed in this study. Data sharing is not applicable to this article.
